# The Role of Emotion Regulation and Executive Functioning in the Intervention Outcome of Children with Emotional and Behavioural Problems †

**DOI:** 10.3390/children10010139

**Published:** 2023-01-11

**Authors:** Blossom Fernandes, Mark Wright, Cecilia A. Essau

**Affiliations:** 1 Department of Health Services Research and Policy, Faculty of Public Health and Policy, London School of Hygiene and Tropical Medicine, London WC1H 9SH, UK; 2 School of Psychology, University of Roehampton, London SW15 4JD, UK; 3 School of Humanities and Social Science, University of Brighton, Brighton BN1 9PX, UK

**Keywords:** emotional and behavioural problems, emotion regulation, executive function

## Abstract

Emotional and behavioural problems are closely associated with impairments in regulating emotions and in executive functions (EF). To examine this further, the aim of the present study was to determine whether EF and emotion regulation at baseline would predict emotional and behavioural problem scores post-intervention, and further explore the extent to which emotion regulation mediates these outcomes. Participants were 41 primary school children who exhibited emotional and/or behavioural problems, aged 8 to 11 years. All the children completed measures of emotional and behavioural problems, cognitive emotion regulation, anxiety symptoms, and performed two experimental tasks to measure working memory and response inhibition before and after participating in a transdiagnostic Cognitive Behaviour Therapy-based programme, “Super Skills for Life” (SSL), and at 3-months follow-up. Results revealed significant reduction in the use of maladaptive emotion regulation strategy catastrophising and other blame following the intervention. Additionally, EF and emotion regulation was associated with outcomes for emotional problems and conduct problems. More specifically maladaptive emotion regulation strategy such as catastrophising and other blame was closely related with self-reports of emotional problems, likewise other blame, was also linked with scores of conduct problems. This study provides preliminary empirical support for EF and emotion regulation in predicting outcomes of emotional and behavioural problems in children following intervention.

## 1. Introduction

The number of children exhibiting emotional and behavioural problems is significantly higher than previously thought. One in five children in the UK have emotional and behavioural problems [1] and these problems tend to emerge in children as young as six years old [2]. Emotional and behavioural problems in children have been closely linked with difficulties in regulating emotions [3,4,5,6]. As argued by Aldao et al. [3], maladaptive emotion regulation strategies, such as rumination, could be directly linked to the development of emotional and behavioural problems. Likewise, executive function (EF) impairments are also associated with emotional and behavioural problems [7], with Warren and colleagues [7] claiming that depressive mood and anxiety are linked to disruptions in maintaining task goals, and importantly, that EF impairments contribute towards the maintenance of emotional disorders.

EFs are cognitive processes, which allow individuals to organise, plan and set goals. These functions are supported by core underlying processes such as working memory, inhibition and cognitive flexibility/set shifting [8]. Specific EF processes are associated with emotional disorders such as anxiety. In a study by Toren et al. [9], children with an anxiety disorder performed poorly on EF tasks compared with children without this disorder [9]. Similarly, young adults with anxiety and depression had significantly lower scores on EF tasks compared with healthy controls [10]. Specific EFs are further associated with emotional problems and the ability to use effective emotion regulation strategies to deal with stressful situations [11]. For example, O’Rourke et al. [11] found that EF impairments were linked to maladaptive coping strategies (e.g., rumination), which, in turn, was related to anxiety. With research showing maladaptive emotion regulation linked to emotional disorders such as depression then predicting impairments in episodic memories in adulthood [12].

Pruessner and colleagues [13] suggest that for emotion regulation strategies to be implemented and maintained core EF functions are necessary, for example, cognitive flexibility and control would allow for effective emotion regulation strategy selection and application. This level of processing then requires effective working memory [13]. Hence, children and adolescents who often use maladaptive emotion regulation strategies, also report anxiety and depression [14,15,16]. In contrast, research focusing on conduct problems in children report that EF deficits may not always be present in those exhibiting conduct problems, however, they are more likely to exhibit maladaptive emotion regulation strategies [17,18]. For example, a study by Tajik-Parvinchi et al. [17] showed that children with cognitive difficulties experience greater maladaptive emotion regulation, which then predicts both emotional and behavioural problems [19].

Young people with emotional and behavioural problems tend to exhibit difficulties in regulating emotions [3,4,5,6] and have EF impairments [20,21]. In a study involving adults, cognitive behavioural therapy (CBT) augmented with executive skills training sessions have led to a significant improvement in EF skills including working memory, inhibition and set shifting tasks [22]. Mohlman [20] argues that CBT may be effective in enhancing EFs of those with emotional disorders, suggesting that EF performance could be improved following interventions based on principles of CBT (i.e., social competence and cognitive restructuring). Similarly following a mindfulness based CBT programme, individuals were found to exhibit improved EF skills and reduced difficulties with emotion regulation [23]. Huang et al. [21] reports that reduced emotional interferences reduces the cognitive load, thereby leading to more effective EF.

For children and adolescents with emotional problems, such as anxiety and depression, CBT is the treatment of choice [24,25,26] and is recommended by the National Institute for Health and Care Excellence in England (NICE; 2011). CBT programmes, such as Super Skills for Life programme [27], allows children to recognise anxious feelings and physiological reactions, and clarify thoughts (e.g., unrealistic or negative attributions and expectations) that occur during anxiety provoking situations. CBT utilises the concept of cognitive restructuring, which may be closely associated with EF processes [28]. The ability to amend cognitive patterns requires underlying EF processes to function effectively and then efficiently implement emotion regulation strategies [25]. This ability to supress a dominant response and implement an adaptive strategy is considered an important aspect of CBT [29]. Following CBT, evidence has shown a significant reduction in behavioural problems in boys aged 6–11 years, alongside improvement in emotion regulation skills [30]. Research also shows that CBT is effective in improving emotion regulation abilities among young people with emotional problems; for example, children and adolescents with anxiety disorders have been shown to have increased emotional awareness and reduction in emotional dysregulation following CBT [31].

Using the transdiagnostic CBT-based programme Super Skills for Life (SSL), children and adolescents with emotional problems in Mauritian residential care institutions showed a significant increase in inhibitory control, an increase in adaptive (e.g., positive reappraisal) and decrease in maladaptive (e.g., rumination) emotion regulation strategies; and fewer internalising and externalising symptoms [32]. Several other studies have highlighted the effectiveness of the SSL programme in improving emotional and behavioural problems in children and adolescents [27,33,34,35]. These studies demonstrate that emotional problems following the SSL programme significantly reduced; moreover these effects were maintained 12 months post intervention [33].

The aim of the present study was therefore to examine the extent to which EF performance and emotion regulation at baseline would predict emotional and behavioural problem scores at post-intervention, and to examine whether the emotion regulation strategies mediate these outcomes. Specific emotion regulation strategies (e.g., other blame) have been found to mediate the relationship between emotional and behavioural problems [35], however studies that examine whether emotion regulation mediates the relationship between EF and emotional and problem behaviours are few, and have shown mixed results. For example, one study reported little effect of emotion regulation on emotional problems following CBT [36], whereas for behavioural problems, psychosocial interventions have shown to improve emotion regulation [30]. This study therefore aimed to examine the extent to which EF predicted emotional and behavioural problems and the emotion regulation strategies that mediate these outcomes. Based on previous studies of EF [19,22,32], firstly we expect a significant reduction in reported emotional, and behavioural problems after the intervention. Secondly, we expect a significant increase in the use of adaptive emotion regulation strategies, and a decrease in maladaptive emotion regulation strategies post intervention. Finally, for EF tasks we also expect to see links to post intervention outcomes with emotional and behavioural problems.

## 2. Method

### 2.1. Participants

Participants were 41 school children (*n* = 30 male pupils, *n* = 11 female pupils), aged between 8 and 11 years (*M*_age_ = 9.53, *SD* = 1.09). The schools were state funded schools, located in ethnically diverse neighbourhoods in Southwest London, the number of pupils from diverse ethnic groups attending the school was above national average (Department for Education, UK, 2017). The children were referred by their class teachers from four primary schools in Southwest London because they exhibited emotional and behavioural problems. Research shows that teachers can act as key informants in assisting with identifying emotional and behavioural problems [37,38].

### 2.2. Procedure

Following approval of ethics from the university psychology ethics committee, the schools were approached via email. Once the school’s head teachers confirmed their participation in the study, the SENCOs and deputy heads were then approached by the head teacher to liaise with the researcher. As this was an opt-out study, parents were sent letters informing them of the nature of the study and giving them the opportunity of 2 weeks to inform the teachers who selected the participants if they wish their child to be excluded from the study.

**Figure 1:**
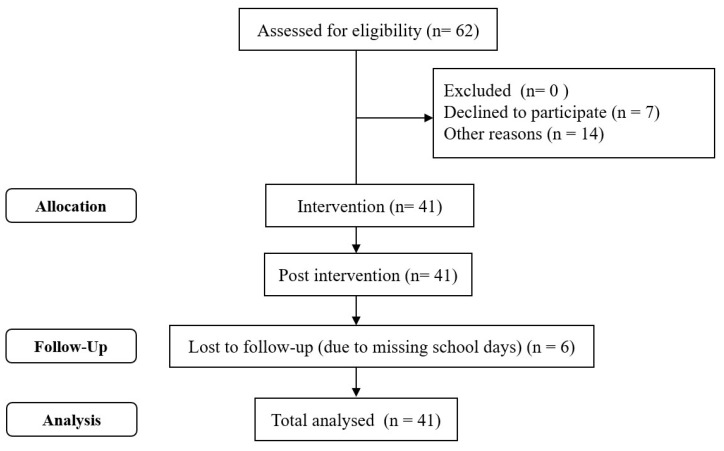
Intervention Flow.

Prior to the intervention, children completed baseline tasks and questionnaires which were also completed immediately post intervention and at follow up 3 months later. They were informed about their participation in the programme and the lead researcher of this study provided instructions and supported those children who required any further assistance with the completion of the tasks or questionnaires. The tests were conducted during school hours. The computerised tasks were published online using the millisecond software; the task sequence was set beginning with the dot probe task, followed by the questionnaires which were also completed online as published on the Qualtrics website. The whole procedure ended with testing participants on the digit span task. At the start of the programme the children were all provided with a workbook and were informed that they would be taught specific skills to cope with challenging and anxiety provoking situations. In total 5 children missed one session and only 1 child missed 3 sessions due to absence from school as a result of illness, this child was however updated with an individual session. The flow diagram of the study can be seen in Figure 1.

### 2.3. Super Skills for Life Programme

The Super Skills for Life (SSL) [24,27] is a psychosocial programme for the prevention of anxiety and depression in young people. It is based on five core principles: (i) it uses a transdiagnostic approach by targeting common core risk factors such as low social skills and self-esteem; (ii) it is based on the principles of CBT to help children develop skills to cope with anxiety-provoking situations; (iii) it uses video feedback with cognitive preparation to assist children with enhancing their self-perception; (iv) it relies on behavioural activation, by encouraging children to increase their activity levels; and (v) it involves teaching children skills to use during social interactions. SSL was delivered by the lead author and other graduate students with previous experience of providing emotional support to children and adolescents in a non-clinical setting. The facilitators received an intensive one-day workshop by the senior author (CAE) of SSL [27]. All the facilitators were given a leader’s manual which included a detailed outline of each session of the SSL.

### 2.4. Implementation of SSL

SSL consists of eight sessions which were implemented twice a week, for the duration of four weeks. Each session lasted approximately 45 min, with an average of six children per group. The commencing session was an opportunity for the children to introduce themselves and understand the purpose of being part of the programme; this was followed immediately by requesting the children present a piece about themselves for a 2-min video recording in their intervention group. The following session involved going over the videos as a group and discussing positive features and aspects of each individual recording, and areas of improvement were discussed from the child’s perspective. For the remaining sessions, children were introduced to topics such as ‘recognising feelings’, thoughts, and the link between feelings, thoughts, and behaviours. Midway through the intervention, a session was devoted solely to teaching the children relaxation techniques, by helping them identify different muscles and possible ways to relax them. The following sessions were then focused on topics such as social skills and problem-solving steps during social conflicts. Most of the sessions began with low intensity physical warm up to increase core and muscle temperature. Similarly, another component of the programme involved dedicating 5–10 min of the session to structured play; these were games which required following instructions and being physically active. The programme also required children to complete home tasks involving activities that were taught in session and which can be applied to their usual setting of playground or home.

### 2.5. Measures

Children completed a set of questionnaires and two experimental tasks before and after the intervention, and three months following the end of the intervention.

### 2.6. EF Tasks

Digit span, a subscale of the Wechsler Intelligence Scale for Children (WISC III; Wechsler, 1991), was used to measure working memory. The forward and backward digit span tests were used, and the participants were required to recite a sequence of digits after the sequence was verbally presented. Following the same principle, participants were then required to repeat the sequence in reverse order for the backward digit test, respectively. High scores on this task indicate greater performance.

**Figure 2:**
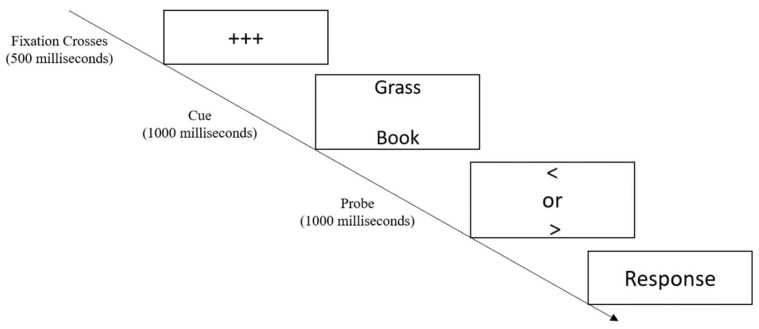
Dot Probe Task.

Dot Probe Task [39] was used to measure inhibition which is one of the core underlying processes of EF [8,40]. The task required the participants to identify a non-emotional probe which was a word, which can appear in one of two spatial locations. Immediately prior to the probe presentation, emotional and non-emotional words appeared simultaneously in two separate locations (see Figure 2). The mean percentage of correct responses was determined for the block of trials for each participant as a measure of inhibition, higher scores indicate greater performance.

### 2.7. Questionnaires

Strengths and Difficulties questionnaire (SDQ) [41] was used to measure emotional and behavioural symptoms. Its 25 items are set on a 3-point Likert scale, ranging from 0 (Not True) to 3 (Certainly True). This scale can be categorised into five subscales: emotional symptoms, conduct problems, hyperactivity, peer problems and pro-social behaviour. The mean scores of participants at baseline were close to average, in line with UK cut off scores. To get the total of the difficulties score, all the subscales except for the pro-social behaviour subscale were added up; the higher the scores, the greater the difficulties. For SDQ pre intervention Cronbach’s alphas in the current study ranged from 0.19 to 0.41.

Cognitive Emotion Regulation Questionnaire (CERQ) [14] was used to measure the use of emotion regulation strategies. It consists of 18 items, which are rated on a 5-point Likert scale ranging from 1 (almost never) to 5 (almost always) in response to how often each of the following strategies are used. The items are categorised into 9 separate emotion regulation strategies: refocus on planning, putting into perspective, acceptance, positive refocusing, positive reappraisal, self-blame, other blame, rumination, and catastrophizing. Self-blame, other blame, rumination and catastrophising are often considered maladaptive strategies, whereas refocus on planning, putting into perspective, acceptance, positive refocusing, positive reappraisal are putatively adaptive strategies. The internal reliability scores for CERQ pre-intervention scores in this study for each strategy ranged from 0.19 to 0.64.

The Screen for Child Anxiety Related Emotional Disorders (SCARED) [42] was used to measure symptoms of common anxiety disorders in children and adolescents. It consists of 38 items which can be categorised into five subscales: somatic/panic, generalized anxiety, separation anxiety, social phobia, and school phobia. Participants are required to indicate the likelihood of experiencing each symptom on a 3-point scale: 0 (almost never), 1 (sometimes), and 2 (often). Reliability scored for SCARED ranged between 0.55 to 0.80.

## 3. Results

### 3.1. Preliminary Analysis

The mean of SDQ, SCARED, CERQ and EF variables for the children at pre- and post-intervention, and at 3-month follow-up are reported in Table 1. A series of one-way repeated measures analysis of variance (ANOVA) were conducted to examine the difference in outcome variables at pre- and post-intervention and at follow-up.

The ANOVA results showed a significant effect of time on maladaptive emotion regulation strategies catastrophising and other blame (Table 1). Post-hoc analysis of Bonferroni further showed significant differences at follow-up for catastrophising (*p* < 0.001) and other blame (*p* < 0.05). No significant main effect of time was found for adaptive emotion regulation strategies acceptance, planning, positive reappraisal, positive refocusing and rumination.

For measures of EF, there was a significant effect of time on performance for forward digit span task (Table 2). These effects were not observed for backward digit span task. There was a reduction in reports of self-blame, emotional and conduct problem scores (based on SDQ) and anxiety scores (based on SCARED), however, these reductions were not significant; and an increase in performance on the inhibitory control task, dot probe (*p* = 0.06) was noted.

### 3.2. Predictors and Mediators of Treatment Outcomes

Regression analysis was carried out to examine the extent to which EF and emotion regulation strategies at baseline would predict emotional and behavioural problem scores at post-intervention. The results showed that EF and emotion regulation strategies predicted outcomes for emotional problems (*F* (8, 27) = 2.35, *p* < 0.05, *R*^2^ = 0.46) and conduct problems (*F* (8, 28) = 2.48, *p* < 0.05, *R*^2^ = 0.47). EF and emotion regulation strategies also predicted hyperactivity (*F* (4, 27) = 2.89, *p* < 0.05, *R*^2^ = 0.41), suggesting that EF and maladaptive emotion regulation strategies at pre-intervention significantly predicted emotional and behavioural problems following the intervention.

EF and emotion regulation strategies at pre-intervention was found to significantly predict emotional problems, conduct problems and hyperactivity post intervention; thus, a further mediation analysis was conducted (Baron and Kenny, 1986) to examine whether the links between EF and emotional problems, conduct problems, and hyperactivity are mediated through emotion regulation strategies. These results showed that there was a significant indirect effect of EF on emotional problems through the maladaptive emotion regulation strategies of catastrophising and other blame (Table 3). There was also an indirect effect of EF on conduct problems through the maladaptive emotion regulation strategy other blame (Table 4). No further indirect effects were observed.

## 4. Discussion

The objective of the present study was to examine the impact of EF and emotion regulation on emotional and behavioural problem scores following a CBT based intervention among children in a typical school setting, as well as examining the extent to which emotion regulation mediate these outcomes.

Our first hypothesis that scores of emotional and behavioural problems would be reduced was supported. As reported by previous findings [24,27,33], participation in SSL led to reduction in emotional problems. In Essau et al.’s study [22], significant reductions in problem behaviours are found six months after the intervention. Similarly, these reductions could be observed 12 months after the intervention [43].

In line with the findings of Ramdhonee-Dowlot et al. [29], our second hypothesis that maladaptive emotion regulation strategies would be reduced was supported in that the maladaptive emotion regulation strategies of catastrophising and other blame were significantly reduced at follow-up assessment. Alongside this, our findings showed that emotion regulation significantly predicted emotional problems, providing further support to the argument that specific emotion regulation strategies are prominently and habitually employed in children with anxiety and depression [44]. More specifically these results show that the emotion regulation strategy other blame at pre-intervention is linked to conduct problems; supporting previous research that report conduct problems are consistently linked with poor emotion regulation [18]. However, there were no significant increases in the use of adaptive emotion regulation strategies, as was expected; this can be explained to some extent, by the mixed findings in literature where adaptive emotion regulation may not always increase following intervention [45]. Even though emotion regulation can be affected following intervention, this change is often dependent on the disorder and age of participants, especially as there are shift in patterns of emotion regulation use during development [40].

Our final hypothesis that EF at baseline would be associated with post intervention outcomes were also supported. Our results support the effect of emotion regulation and EF at pre intervention in predicting outcomes of emotional and behavioural problems post intervention [46]. Baseline EF is associated with emotional problems, but also predict responsiveness to treatment, emphasising the importance of EF in treatment outcomes [46]. Moreover, participating in SSL led to a significant improvement in EF (working memory and inhibition) in that the scores of forward digit span increased post-intervention and at follow-up. These results are similar to the findings reported among children [32] and adults [22,23]. The significant changes observed in EF skills in this study might have contributed to the successful control of emotional problems and maladaptive emotion regulation, as EF is mediated by the prefrontal cortex [47]; abnormalities and variation in this region is likely associated with emotional problems such as anxiety [7,48].

## 5. Strengths and Limitations

A major advantage of the present study was the use of a transdiagnostic CBT-based intervention (SSL) that targets the key risk factors (i.e., EF and emotion regulation) associated with emotional and behavioural problems. The intervention was delivered in a school setting, which may benefit families who otherwise face barriers accessing appropriate services for their child or fear of their child being stigmatised [49]. Furthermore, unlike numerous studies in intervention research, this study used both self-report questionnaires and task-based indicators of outcomes; these tasks included digit span and dot probe task to measure working memory and inhibition.

There are however, methodological limitations which need to be considered when interpreting our findings. The low Cronbach’s alpha scores may have impacted the effect sizes observed; thus, the results should be interpreted with caution. The repeated use of the same task could also have influenced some level of improvement in the participants, therefore future studies could focus on the sequence of tasks to reduce learning effects. Additionally, the absence of a control group could have inflated our findings [50]. Furthermore, a short follow-up period (3 months) and a small sample size could have also affected the results. Finally, the participants were referred by their teachers as having emotional and behavioural problems and subsequently may have affected the generalisability of these results to a clinical population. Children who are identified as having emotional and behavioural problems by their teachers, have been found to be at a higher risk of behavioural problems [51].

## 6. Conclusions

This is the first study to our knowledge to further investigate the mediating effect of emotion regulation and the durability of EF in maintaining positive outcomes for children with emotional and behavioural problems. It would be informative to include a control group to test the effects of practice on the EF tasks. However, from this SSL intervention participants may have been able to derive increased benefits (i.e., enhanced engagement in daily activities, improved functioning in novel situations). Future trials are needed to identify and compare the role of emotion regulation in different groups. Further studies would also benefit from a vast array of EF tasks measuring both cognitive flexibility and affective decision making. These limitations notwithstanding, children who participated in this SSL programme showed a reduction in the maladaptive emotion regulation strategies other blame and catastrophising. Taken together, these results suggest that the outcomes following SSL intervention may be associated with reduction in difficulties in EF and maladaptive emotion regulation of strategy use for children exhibiting behavioural and emotional problems in the classroom. These factors should be considered in interventions aimed at children’s mental health following the coronavirus pandemic lockdown measures.

## Figures and Tables

**Table 1:** Mean differences in SDQ, SCARED, and CERQ pre- vs. post SSL intervention.

	Pre-Intervention Mean (SD)	Post-Intervention Mean (SD)	3-Month Follow Up Mean (SD)	*F*	*Ƞ^2^*
*SDQ*					
Emotional problems	4.24 (2.37)	3.08 (2.77)	3.10 (2.16)	2.45	0.08
Conduct problems	3.37 (2.68)	3.11 (2.20)	2.90 (1.81)	0.30	0.01
Hyperactivity	4.48 (2.32)	4.14 (2.14)	4.48 (2.13)	0.77	0.01
Peer problems	3.25 (2.37)	2.80 (1.94)	2.67 (1.65)	0.74	0.01
*SCARED*					
Panic disorder	6.56 (4.72)	6.14 (5.99)	4.40 (4.46)	1.02	0.03
Generalised anxiety disorder	5.92 (3.50)	5.51 (4.56)	5.25 (4.01)	0.21	0.01
Social Anxiety	6.04 (3.63)	5.64 (3.85)	5.10 (3.80)	0.83	0.02
Separation anxiety	6.44 (3.70)	5.68 (4.32)	5.00 (4.10)	0.64	0.01
School avoidance	1.53 (1.46)	1.81 (1.77)	1.45 (1.35)	0.44	0.01
*CERQ*					
Self-blame	5.37 (1.80)	4.52 (1.95)	5.57 (2.11)	2.62	0.05
Rumination	5.97 (2.21)	5.18 (2.65)	4.86 (1.93)	1.91	0.04
Catastrophising	5.84 (2.23)	5.66 (2.62)	3.95 (1.69)	4.10 **	0.08
Other blame	5.05 (2.36)	4.31 (1.98)	3.76 (1.37)	2.96 *	0.06
Acceptance	5.65 (2.32)	5.09 (1.80)	5.67 (2.08)	0.81	0.02
Positive refocusing	5.38 (2.42)	4.85 (2.30)	4.23 (2.02)	1.74	0.04
Planning	4.86 (2.22)	4.42 (2.26)	4.61 (2.15)	0.72	0.01
Putting into perspective	5.84 (2.23)	5.65 (2.62)	3.95 (1.68)	5.08 **	0.10
Positive reappraisal	5.81 (1.90)	5.02 (2.33)	5.19 (2.16)	0.26	0.01

Note. ** *p* < 0.01; * *p* < 0.05; SDQ = Strengths and Difficulties Questionnaire; SCARED = Screen for Child Anxiety Related Emotional Disorders; CERQ = Cognitive Emotion Regulation Questionnaires.

**Table 2:** Mean differences for EF tasks pre- vs. post SSL intervention.

	Pre-Intervention Mean (SD)	Post-Intervention Mean (SD)	3-Month Follow Up Mean (SD)	*F*	*Ƞ^2^*
Forward digit span	8.85 (2.50)	9.17 (1.98)	9.88 (2.14)	6.28 **	0.21
Backward digit span	3.47 (1.43)	4.10 (2.19)	4.12 (1.88)	2.78	0.11
Dot probe task	66.30 (22.24)	69.78 (18.53)	95.53 (97.52)	1.45	0.07

Note. ** *p* < 0.01; EF = executive functions.

**Table 3:** Results of mediation analysis summary of maladaptive emotion regulation strategies, inhibition, working memory pre-intervention predicting emotional problems. (*N* = 41).

	Inhibition (Dot Probe)			Working Memory (Digit Span)		
*Variable*	*b*	*SE*	*t*	*b*	*SE*	*t*
Catastrophising	−0.39	0.21	−1.80	−0.49	0.19	−2.53 *
Other blame	0.63	0.19	3.21 *	0.64	0.17	3.73 **
Rumination	0.15	0.19	0.81	0.15	0.17	0.89
Self-blame	0.23	0.23	1.01	0.19	0.20	0.93
*R^2^*		0.34			0.44	
*F*		2.70 *			3.98 *	

Note. ** *p* < 0.001, * *p* < 0.05.

**Table 4:** Results of mediation analysis summary of emotion regulation strategies, inhibition, working memory pre-intervention predicting conduct problems. (*N* = 41).

	Inhibition (Dot Probe)			Working Memory (Digit Span)		
*Variable*	*b*	*SE*	*t*	*b*	*SE*	*t*
Catastrophising	0.11	0.20	0.52	0.26	0.19	−1.34
Other blame	0.42	0.18	2.41 *	0.38	0.17	2.17 *
Rumination	−0.25	0.19	−1.28	−0.26	0.19	−1.34
Self-blame	−0.12	23	−0.50	−0.14	0.28	2.14
*R^2^*		0.58			0.63	
*F*		2.89 *			3.45 *	

Note. * *p* < 0.05.

## Data Availability

The data presented in this study are available on request from the corresponding author.
